# Do village-level normative and network factors help explain spatial variability in adolescent childbearing in rural Honduras?

**DOI:** 10.1016/j.ssmph.2019.100371

**Published:** 2019-02-04

**Authors:** Holly B. Shakya, John R. Weeks, Nicholas A. Christakis

**Affiliations:** a Center on Gender Equity and Health, Division of Infectious Diseases and Global Public Health, University of California San Diego, La Jolla CA, USA; b Department of Geography, San Diego State University, San Diego CA, USA; c Department of Sociology, Yale University, New Haven CT, USA

**Keywords:** Social norms, Social networks, Adolescent fertility, Honduras, Spatial analysis, Social cohesion

## Abstract

Adolescent childbearing rates are higher in Central America than almost anywhere else. However, in this research we discovered that adolescent childbearing exhibits variability from one village to another, and we might discover factors associated with this spatial variability that can help us understand key characteristics underlying the pattern of early childbearing. To do this, we assessed the village-level normative and network factors associated with adolescent birth (birth taking place before age 20 years) in rural Honduras and evaluated the geographic dispersion of these patterns. We used full population data from 24,937 people in 176 villages (81% of the eligible population) to assess prevalence and patterns of adolescent childbearing among women. We modeled the predictors of adolescent births among women younger than 21 years. After accounting for individual demographic characteristics, one of the strongest predictors of adolescent birth within the population was village-level collective norms about the acceptability of adolescent childbearing, based on aggregating normative measures from the entire population. The proportion of women in the village who had given birth as an adolescent was also strongly associated with an individual girl's likelihood of having given birth as an adolescent. We used full village-level network analyses to calculate social cohesion within the village. Normative pressure was strongly associated with the likelihood of an adolescent birth in villages with high cohesion (high network density) and was not associated or had a weak association in villages with low cohesion. On the other hand, the longer a girl had lived in the village, the stronger the association between the overall proportion of women in that village who gave birth as adolescents and the girl's own likelihood of having done so. Spatial analyses suggest that levels of adolescent births vary spatially across villages, as do the village-level normative factors associated with them.

## Highlights

• Honduras has very high rates of adolescent fertility, especially among rural poor.
• We use data from 176 villages in Honduras (N = 24,956) including social networks.
• Village norms (descriptive, injunctive) are associated with adolescent fertility.
• The impact of norms varies by time in village, and village level social cohesion.
• There are geographic groups of villages with high norms and high adolescent fertility.

## 1 Introduction

**Fig. 1:**
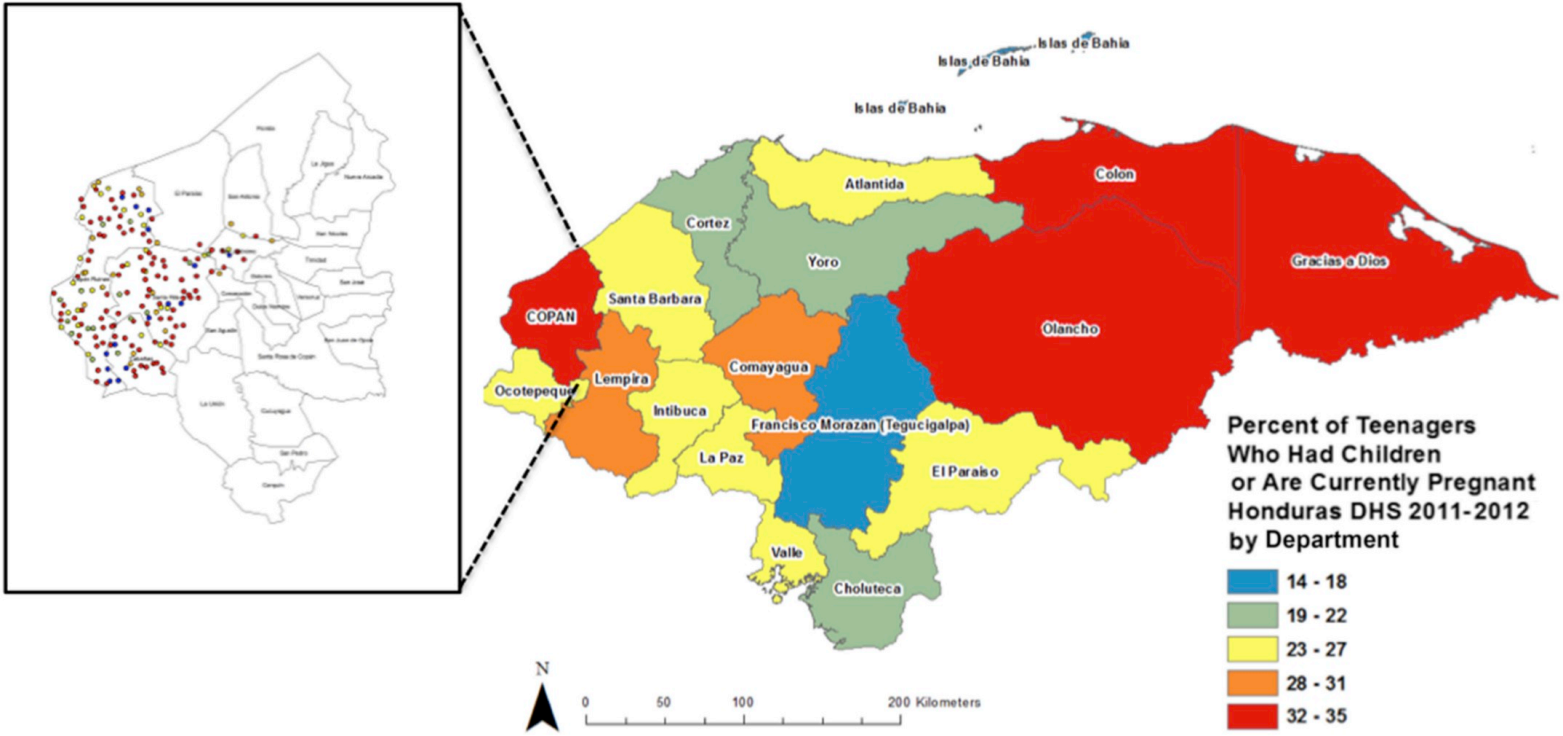
Teenage pregnancy in each Department of Honduras and in the villages in the Department of Copán, from which data were collected for this research. DHS indicates Demographic and Health Survey.

Adolescent fertility rates are high in Latin America, a significant concern for the region given that giving birth during adolescence has been associated with a wide array of subsequent physical and mental health problems, as well as enduring socioeconomic issues that may persist into later life (Angelini & Mierau, 2018; Chen et al., 2007; Ganchimeg et al., 2014; Sagili, Pramya, Prabhu, Mascarenhas, & Rani, 2012). Within the context of Latin America, and Central America more specifically, Honduras has one of the highest rates of adolescent childbearing, with 24% of adolescent girls between the ages of 15 and 19 years either already mother or currently pregnant, according to data from the most recent Honduras Demographic and Health Survey (DHS) in 2011–2012 (USAID, 2016). However, these rates are not uniformly high throughout the country. Fig. 1 shows that the percentage of teens adolescents girls who had a child or were pregnant at the time of the DHS interview is lowest in the capital city of Tegucigalpa (still twice as high as in Accra, the capital of Ghana, for comparison). The highest levels are found in the eastern and western extremes of the country, including Copán, the department (or province) in which the study site for this analysis is located, where one in three adolescent girls was pregnant or already had a child in 2011 or 2012. Rural residence is also a strong predictor of adolescent birth, with the 2011 Honduras DHS showing that 15% of adolescent girls aged 15–19 years became mothers in urban areas, compared with 23% in rural areas (DHS Program, 2018).

Once adolescent girls become mothers, disadvantage can become enhanced or entrenched and is often passed on to their own daughters. This cyclical process is inextricably linked to a long list of individual risk factors, most of which are associated with a life of disadvantage. Determinants of adolescent childbearing in Latin America include living in poverty (Goicolea, Marianne, Öhman, & San Sebastian, 2009), experiencing physical or sexual abuse as a child (Goicolea et al., 2009; Pallitto & Murillo, 2008), family disruption including parental migration (Goicolea et al., 2009; Guijarro et al., 1999), fatalistic attitudes about the future (Goicolea, 2010), experiencing intimate partner violence as an adolescent (Pallitto & Murillo, 2008), and low levels of parental education (Guijarro et al., 1999). Latin American girls that have babies as adolescents are more likely to enter into unstable romantic partnerships or unions as a result of the birth and eventually have children with multiple partners, often raising their children without a father or stable financial support (Grace & Sweeney, 2014; Schmeer & Hays, 2017).

Although individual risk factors can be strongly predictive of adolescent birth, higher-level interpersonal and community factors are an important part of the equation, albeit one that is studied less frequently. Previous research has shown that the risk of adolescent childbearing within Latin America decreases within communities and families that are socially cohesive, that emphasize respect for family, and that hold strong norms against early pregnancy (Denner, Kirby, Coyle, & Brindis, 2001; Guijarro et al., 1999; Martyn, Darling-Fisher, Smrtka, Fernandez, & Martyn, 2006). Girls who live in communities with a strong cultural emphasis on motherhood, conversely, are at higher risk of adolescent childbearing (UNICEF, 2014). Much of the previous research on these contextual factors has been qualitative, as most population-level surveys such as DHS lack contextual level and social normative measures. Because there is little data on these factors, the degree to which contextual factors are associated with adolescent birth is still unclear.

### 2 Theoretical perspective

#### 2.1 Social norms

Communal normative pressures appear to be important in helping to regulate adolescent childbearing within the Latin American context. Social norms are established standards of behavior maintained by a society. They serve to encourage and enforce what the group deems to be appropriate behavior, while discouraging and punishing behavior that is deemed to be inappropriate (Schaefer, 2015). Social norms operate as part of a system of social control (Macionis, 2015). Research on social norms has highlighted the difference between “descriptive norms,” which are prevalent behaviors within a community but not necessarily socially reinforced, and “injunctive norms”, which are enforced within a community through sanctions—either positive sanctions for behaving within normative expectations or negative sanctions for normative violations (Cialdini, Kallgren, & Reno, 1991; Lapinski & Rimal, 2005). The rich body of research on *diffusion of innovations* has mostly focused on descriptive norms, which operate through a mechanism of social learning (Mackie, Moneti, Denny, & Shakya, 2012). Descriptive norms are ideally measured by asking individuals what they believe others in their community are doing. However, these norms are often inferred by aggregating the proportion of people who engage in a behavior at a higher level, such as a village or school. Injunctive norms can be proscriptive (what you should not do) or prescriptive (what you should do) (Janoff-Bulman, Sheikh, & Hepp, 2009) and are enforced through direct social influence as a means of social control. Although behaviors that are regulated through injunctive norms are frequently observable and can therefore also operate as descriptive norms, descriptive norms can also reflect behaviors that are simply tolerated rather than directly enforced through social control.

Adolescent childbearing is socially complex, so reduction strategies that focus solely on individual-level determinants are often unsuccessful (Bearinger, Sieving, Ferguson, & Sharma, 2007; Speizer, Magnani, & Colvin, 2003). Adolescent childbearing, like most behaviors, can be influenced by descriptive norms, injunctive norms, or both. There are, of course, two types of behaviors involved in adolescent childbearing: 1 sexual intercourse and 2 birth control (or the lack/failure thereof). In contrast to places like rural Cameroon, in which adolescent childbearing is the expected result of adolescent marriage and is a socially reinforced expectation to preserve family honor and prevent pregnancy out of wedlock (Shakya, Mackie, Nkwi, Pererya, & Cislaghi, 2018), adolescent childbearing in Latin America is often the result of unregulated sexual activity outside of marriage (Laplante, Castro-Martín, Cortina, & Fostik, 2016). Tolerance for adolescent childbearing in Latin America is further compounded by a larger societal injunctive norm that places a high value on motherhood for women (Schmeer & Hays, 2017; UNICEF, 2014).

#### 2.2 Spatial dependency

An important clue regarding the presence of social norms is spatial variation in the prevalence of behaviors. Spatial demographers assume that place is an important determinant of attitudes and behaviors, because geographic features can inhibit or facilitate behaviors (eg, distance to a health clinic) and perhaps more importantly because it is through *spatial* clustering of people that clustering of norms typically occurs (Uthman, 2008; Weeks, 2004, 2015). If an outcome of interest is *spatially dependent,* it means that people in close proximity to each other will share certain characteristics in common (Weeks, 2015). For example, a recent study of fertility rates among very young adolescents (aged 10–14) in Brazil found significant spatial clusters of adolescent fertility across the country, with high-rate clusters predominant in the northern region and low-rate clusters predominant in the southern, southeastern, and midwestern regions (Borges et al., 2016). *Spatial heterogeneity*, on the other hand, refers to the fact that what might seem like global associations may actually vary by place (Lu, Charlton, Harris, & Fotheringham, 2014; Weeks, 2015) For instance, the relationship between poverty and adolescent fertility may vary geographically depending upon local norms within communities, which may be place-specific.

## 3 Research question

Here we utilize a unique dataset with a complete census of the population from 176 villages in rural Honduras, their social network connections, and spatial data to assess whether village-level normative and social network factors are associated with giving birth before age 18 years, with a focus on delineating the role of individual-level normative factors from those held at the community or village level. Spatial data allow us to determine whether there are spatial patterns of adolescent births and the norms associated with them, an important consideration for programs designed to decrease rates of adolescent childbearing. We hypothesize: 1) in communities in which there are injunctive norms against adolescent childbearing, adolescent girls may be less likely to become mothers; 2) in communities in which a high proportion of women gave birth as adolescents (strong descriptive norms), adolescent girls are more likely to become parents; 3) adolescent girls in more socially cohesive communities are less likely to have given birth as adolescents; 4) normative influence is stronger in more cohesive communities; and 5) these normative patterns are geographically clustered.

## 4 Data and methods

### 4.1 Study population

Our study uses full population census data from the western municipalities of the largely rural Copán department of Honduras (see Fig. 1) to analyze the determinants of adolescent fertility at the village level. Data were collected as part of a randomized controlled trial of social network targeting of a maternal and neonatal health intervention in this area (Kim et al., 2015; Shakya et al., 2017). There are 238 villages located in the study area. The final set of 176 villages for which we have data were chosen based on a combination of population size, accessibility, and safety for a subsequent random intervention program. A full description of the study design and data collection methods are published elsewhere (Shakya et al., 2017). The area was chosen because of the geographic diversity of its villages, population vulnerability to maternal and neonatal health complications, and suitability for data collection. This part of Honduras also has a traditionally high rate of adolescent childbearing (USAID, 2016), making it an ideal location for understanding the distribution of adolescent childbearing.

We completed geographical mapping for 176 villages chosen for the randomized trial located in the municipalities of Copán Ruinas, Santa Rita, Cabañas, and San Jerónimo in the department of Copán, allowing us to gain more precise calculations of the study population and field conditions, including terrain, rainfall, and distances to health facilities. The area is over 200 square miles of rugged mountainous terrain with an estimated total population (in the 176 villages) of 32,800 people older than 12 years (the total population in all four municipalities is approximately 92,000, which includes people not in our selected villages and people outside our age range). We conducted a census in 2016 with 92% of the eligible population, of which 25,032 completed a baseline survey that included sociocentric and behavioral health measures. For the purposes of the fertility analyses, we excluded children younger than 15 years (N = 2577), as they did not complete the full reproductive history and the proportion who had already experienced a birth was miniscule. Individuals who were cognitively impaired and unable to provide consent were also excluded (30). Our final number of participants was 25,032, of which we used data from 22,449 for the fertility analyses reported in this paper.

### 4.2 Network data collection

We used the publicly available Trellis software (http://humannaturelab.net/resources/software/trellis/) to undertake the main survey, which included a battery of “name generator” questions to capture social relationships. In this study, the boundaries of each network were the villages, so that individuals could nominate any individual from within their own village as a social contact. Photographs were taken of all persons from whom data were collected, and they were used to validate the social contacts named by the respondents.

### 4.3 Measures

Our unit of analysis for the descriptive assessment of communal and personal norms regarding adolescent parenthood across gender and generations is all respondents across 176 villages. However, for our statistical analysis of practices, our unit of analysis is restricted to individual women and girls between the ages of 15 and 20 years at the time of the survey, for whom an adolescent birth would have occurred within the last few years (N = 2990). The outcome, adolescent birth, was defined in two ways. Our definition of adolescent motherhood was having had a child before age 20 years, which is consistent with the definition used in DHS and other similar demographic surveys.

#### 4.3.1 Outcome variable: adolescent childbirth

Female respondents were asked whether or not they had ever given birth to a living child. Women who reported having given birth were then asked to provide the birthdates of their last four children. For women with four or fewer children, their age at first birth was calculated as the difference between their date of birth and the date of birth of their first child. For women with more than four children, the age at which they first gave birth was approximated using a question that asked the age at first pregnancy. None of the women in our primary sample population of women aged 15–20 years had more than four children.

#### 4.3.2 Individual demographics

Individual demographic controls included age, marital status, religion, income sufficiency, education, food security, proportion of life lived in the village, and indigenous status. For exact coding of these measures please see the online appendix.

#### 4.3.3 Attitudes and social norms at the individual level

All respondents were asked their personal attitudes regarding the appropriate age for a first birth for women: “At what age is it OK for a girl to have her first baby?” We also asked each respondent about injunctive norms around adolescent birth: “If a girl younger than 18 has a baby, will people in the community think this is good, bad, or neither?” We modeled normative beliefs in support of adolescent birth as a binary variable, “good” or “bad/neither,” as the statistical model showed no difference in the association between “bad” or “neither” with adolescent birth but a strong difference between “good” and “bad/neither.” In this case, coding the variable as continuous would have resulted in an artificial result suggesting a linear relationship.

#### 4.3.4 Village-level factors

Because the unit of analysis is women younger than 21 years, all individual-level variables are specific to that population. However, we constructed the village-level normative and social network factors using the *entire sample of men and women of all ages for each village* to get a comprehensive understanding of the village-level social environment.

#### 4.3.5 Village-level normative factors

We aggregated the means of individual attitudes regarding the appropriate age for first birth (collective attitudes), and the proportion of each village that reported perceptions of norms regarding giving birth before age 18 years as “good” (collective injunctive norms). As a proxy for descriptive norms, which would ideally be measured by asking each respondent what they think is normally practiced, we calculated the proportion of women in the village overall who gave birth before age 20 years (descriptive norms).

#### 4.3.6 Village-level network factors

As part of the network survey, respondents were asked 12 separate questions regarding their social connections within the community, including familial relationships, close personal relationships, economic support, and health advice (See Supplementary Appendix for specific questions). To assess village-level social cohesion, we calculated *density*, a measure of cohesion at the network level, using the comprehensive network constructed from the relationships across all relationship questions and across all members of each village. Density is a measure of the number of identified ties over the total number of possible ties (Valente, 2010). A measure of density provides insight into how closely connected the people are in the village.

#### 4.3.7 Spatial measures

We collected coordinates (x, y) for the approximate geographic center of each village in the dataset. (For details on the geographic data collection see Supplementary Appendix.)

### 4.4 Statistical analysis

As noted above, our primary unit of analysis for our predictive models was individual women between the ages of 15 and 20 years at the time of the survey (N = 2990), with the primary outcome being adolescent birth, or having given birth before age 20 years. All of our analyses were based on multilevel logistic regression, clustering at the village level. A −2 log-likelihood test confirmed significant village-level clustering and the appropriateness of using a multilevel model. Continuous variables, including village-level network measures and proportions, were z-score centered. Our initial models included individual-level factors, and subsequent models included village-level social normative factors controlling for village size. Finally, we ran interactions testing whether village-level normative factors were moderated by village-level cohesion, to determine whether or not cohesion alters the impact of significant village-level normative factors on our outcomes.

#### 4.4.1 Spatial analyses

Despite the overall high level of adolescent childbearing in this rural area of Honduras, there is considerable variability both within and between villages in the likelihood of adolescent birth. Although statistically significant village-level clustering is important evidence that supports the validity of our findings on the association between village norms and the level of adolescent births, it does not answer the question of whether this village-level variation is spatially dependent. In other words, are there clusters of villages with higher or lower rates of adolescent fertility, with associated higher or lower rates of norms in favor of it?

We used the Getis-Ord Gi* hot spot statistical analysis tool to determine the existence of statistically significant spatial clusters of high or low values. As social norms tend to cluster within geographic areas, we conducted a series of spatial analyses to discern to what extent the incidence of adolescent birth and the norms associated with it are spatially significant. Our spatial analyses included hot spot analysis using the Getis-Ord Gi* statistic (within ArcGIS) to examine significant spatial clustering of villages with regard to norms surrounding approval of adolescent birth, as well as the recent incidence of giving birth before age 18 years. We further explored the spatial patterns in our analysis to evaluate the presence of spatial non-stationarity—spatial variation in the regression relationships. In other words, do we find evidence that there are geographic differences in the strength of the association between village-level normative beliefs and the rate of adolescent childbearing in these villages? We used geographically weighted regression to assess possible spatial differences in the association between demographic and normative factors and adolescent birth within the village. We also used the ArcGIS spatial clustering grouping analysis tool, with spatial constraints set to K-nearest neighbors (using a minimum of 8 neighbors), to identify spatially contiguous villages that have similar characteristics with respect to levels of and norms regarding adolescent birth. These could be thought of as “neighborhoods” of similar patterns of adolescent fertility. Note that this is an exploratory tool, but the results can be very suggestive of underlying social processes even if not definitive.

## 5 Results

### 5.1 Descriptive

Adolescent birth is common within this population of women. Across the entire sample of women, including those younger than 21 years, 44% had given birth as an adolescent (see Table 1). Among those women younger than 21 years, the proportion who gave birth as adolescents is 32%, although some of them are still young adolescents who may become mothers but have not yet. Across the sample, the median age given for ideal first birth was 21 years, while the proportion who reported injunctive norms in favor of giving birth before age 18 years was 16%. When we aggregate these measures at the village level, we see marked variation. Across villages the average proportion of women who gave birth as adolescents was 39%, with a range of 6%–73%. Although there is village-level variation in collective attitudes regarding the ideal age for first birth (mean, 21.4 years; range, 19.7–23 years), there was considerable village-level variation in the proportion that report injunctive norms in favor of adolescent birth (mean, 16%; range 2%–43%).

**Table 1:** Descriptive statistics of study population.

	Women aged <21 years	Women aged ≥21 years
	Mean (SD)	Mean (SD)
**Individual factors**^a^		
Age (SD)	17.4 (2)	40.1 (15)
Proportion of total sample who have had a child or are currently pregnant	32%	92%
Age at birth of first child for those who are parents	16 (1.6)	19 (3.8)
Proportion with first child born before age 18 years	24%	33%
Proportion with first child born before age 20 years	31%	44%
Education (0–9)	4.9 (2.1)	2.7 (2.4)
Income sufficiency	2.8 (0.8)	2.5 (0.8)
Proportion who lived their whole life in the village	67%	48%
Proportion whose birth occurred pre–village residence	6%	26%
Attitude towards best age for first birth	21.4 (2.6)	21.2 (2.5)
Proportion who think community believes giving birth before age 18 years is good	17%	16%
	Mean (Range)	
**Village-level proportions and means across villages**		
Proportion in village who report community norms that birth before age 18 years is good	0.16 (0.02–0.43)	
Mean reported best age for first birth	21.4 (19.7–23.0)	
Proportion of women in village who gave birth before age 18 years	0.31 (0.06–0.58)	
Proportion of women in village who gave birth before age 20 years	0.39 (0.06–0.73)	
Village-level density	0.03 (0.01–0.14)	

a For details on coding of demographics, please see online appendix.

### 5.2 Individual-level factors

Our first set of analyses focused on individual girls and women between the ages of 15 and 20 years (N = 2990). Table 2, Model 1 shows individual characteristics that could be reasonably associated with a recent birth event, including demographics and proportion of life lived in the village. For each 1 SD increase in a girl's attitudes about the best age for first birth, the odds of having had a birth before age 20 years decreased by 46% (95% confidence interval [CI], 39%–52%), while girls who reported positive injunctive norms regarding adolescent birth were 32% (95% CI, 5%–67%) more likely to have had a birth before age 20 years. Consistent with previous literature, we find that education is negatively associated with giving birth before age 20 years, as is income sufficiency.

**Table 2:** Individual- and village-level factors predicting having given birth as an adolescent among women younger than 21 years in rural Honduras.

	Bivariate Analyses			Model 1: Individual			Model 2: Multilevel With Village Factors			Model 3: Interaction			Model 4: Interaction		
	B	SE	P	B	SE	P	B	SE	P	B	SE	P	B	SE	P
Village average norms that birth before age 18 years is good (scaled)	0.23	0.04	<0.001				0.13	0.06	0.02	0.21	0.06	0.00	0.13	0.05	0.02
Proportion of village women with an adolescent birth (scaled)	0.19	0.04	<0.001				0.13	0.06	0.04	0.12	0.06	0.06	0.16	0.06	0.01
Individual belief about appropriate age for first age (scaled)	−0.70	0.05	<0.001	−0.61	0.06	<0.001	−0.61	0.06	<0.001	−0.62	0.06	<2e-16	−0.61	0.06	<0.001
Individual perceived norm regarding acceptability of giving birth before age 18 years	0.57	0.10	<0.001	0.28	0.12	0.02	0.24	0.13	0.06	0.25	0.13	0.05	0.23	0.13	0.06
Education (scaled)	−0.52	0.04	<0.001	−0.31	0.05	0.00	−0.34	0.05	0.00	−0.34	0.05	0.00	−0.33	0.05	0.00
Income sufficiency (scaled)	−0.19	0.04	<0.001	−0.12	0.05	0.02	−0.14	0.05	0.01	−0.14	0.05	0.01	−0.13	0.05	0.02
Food security (scaled)	0.16	0.04	<0.001	0.12	0.05	0.02	0.10	0.05	0.05	0.10	0.05	0.06	0.10	0.05	0.09
Religion ref Catholic															
No religion	0.52	0.11	<0.001	0.27	0.15	0.07	0.23	0.15	0.13	0.21	0.15	0.16	0.23	0.14	0.08
Religion Protestant	0.16	0.09	0.07	0.23	0.11	0.04	0.20	0.11	0.08	0.16	0.11	0.16	0.20	0.11	0.32
Proportion of life in village (scaled)	−0.60	0.04	<0.001	−0.45	0.05	0.00	−0.45	0.05	<0.001	−0.46	0.05	<2e-16	−0.46	0.05	0.00
Indigenous	−0.04	0.14	0.78	0.08	0.17	0.63	−0.03	0.18	0.88	0.00	0.18	0.99	−0.06	0.18	0.73
Age (scaled)	1.02	0.05	<0.001	1.07	0.05	<0.001	1.09	0.06	<0.001	1.09	0.06	<2e-16	1.09	0.06	<0.001
Number of households in village (scaled)	−0.14	0.04	0.001				0.05	0.06	0.47	0.02	0.08	0.83	0.04	0.06	0.55
Average education							0.09	0.07	0.18	0.06	0.07	0.34	0.09	0.07	0.20
Distance to main road							−0.11	0.06	0.08	−0.10	0.06	0.06	−0.12	0.06	0.07
Average age							−0.07	0.06	0.25	−0.10	0.06	0.14	−0.07	0.06	0.24
Village-level density < median										0.10	0.14	0.50			
Village norms* village density ​< ​median										−0.30	0.12	0.01			
Proportion of life in village*proportion of village women who gave birth before age 18 years													0.16	0.05	0.00
AIC				2634			2619			2616			2609		
Tjur's D				0.312			0.324			0.324			0.326		

Column 1 shows the results of bivariate analyses. Model 1 is a multivariate logistic regression model showing the association between individual-level demographic and normative factors and adolescent births. Mode 2 combines individual-level and village-level factors using multilevel modeling, clustering on the village. Model 3 shows the results of Model 2 when including the interaction between village-level density and village-level collective injunctive norms. Model 4 shows the results of Model 2 when including the interaction between the proportion of a girl's life spent in the village and the proportion of women in that village who gave birth as an adolescent. Scaled variables are z-score standardized to increase ease of interpretation.

Abbreviations: SE, standard error; AIC, Akaike information criterion.

### 5.3 Village-level normative and network factors

Testing bivariate and multivariate associations at the village level (for more detail see online Appendix 1), we found that both collective injunctive norms in favor of adolescent birth and the descriptive norms (collective adolescent birth) were strongly associated with adolescent birth. Collective attitudes were not significantly associated with adolescent birth.

### 5.4 Individual- and village-level factors: multilevel analysis

In Table 2, Model 2, we show the full multivariate models, including village-level collective injunctive norms in support of adolescent birth and village-level collective adolescent births with the individual-level normative and demographic factors from Table 1. The Aikake information criterion (AIC) decreased 17 points, from 2634 to 2619, a strong indication that Model 2 is a significant improvement over Model 1 (Burnham & Anderson, 2004). Inclusion of the village-level variables did not notably change the coefficients of the individual-level variables, with the exception of individual perception of community support for adolescent birth, which was somewhat attenuated and lost significance at *P* < .05. Both village-level factors retained significance in the full model.

The likelihood that a girl gave birth as an adolescent increased by 13% (95% CI, 2%–28%) for every 1 SD increase in both the proportion of the village that believes the community supports adolescent birth and the village-level proportion of women who gave birth as adolescents. These effect sizes are approximately one-third of what we found for a 1 SD increase in education, which is one of the most well-documented factors associated with adolescent childbearing. When we set model parameters to their means, we find that the predicted probability of a girl having an adolescent birth is 20% (95% CI, 0.16–0.25) when only 10% of the village believes that the community supports giving birth before age 18 years, compared with a probability of 32% (95% CI, 0.23–0.42) when 40% of the village believes that the community supports giving birth before age 18 years.

### 5.5 Normative factors vary by levels of exposure: interaction models

**Fig. 2:**
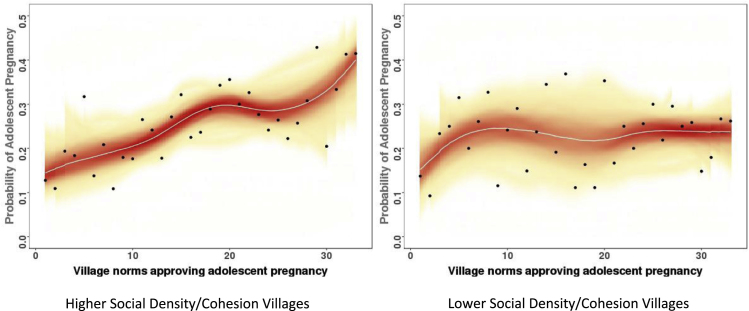
Probability of adolescent pregnancy by the village norms regarding adolescent pregnancy, according to the level of social density/cohesion in the village.

Village-level social network cohesion (network density) did not independently predict the risk of an individual woman having an adolescent birth. However, in an interaction model (Table 2, Model 3), density acts as a significant moderator on the association between collective norms and adolescent births (*P* = .01). Stratifying by cohesion illustrated a lack of association between the proportion of the village who believe the community supports adolescent birth and adolescent birth for low-cohesion villages, and a strong association for high-cohesion villages. Setting all parameters equal, when 40% of the village report that the community supports adolescent birth, the probability of adolescent birth is 35% (95% CI, 0.23–0.49) in high-density villages versus 17% (95% CI, 0.08–0.34) in low-density villages. Fig. 2 further illustrates the differential associations between collective norms and adolescent births for low-density/cohesion and high-density/cohesion villages. To test this further, we tested interactions by transitivity and by limiting our measure of density to strong personal relationships and found the results were the same (not shown).

We did not find a significant interaction between cohesion and the proportion who had given birth as an adolescent. However, we found significant interactions between the proportion of life a spent in the village, the proportion of women in the community who had given birth as an adolescent, and a girl's likelihood of having given birth as an adolescent. The greater proportion of a girl's life spent in the village, the stronger the association between the descriptive norms, the proportion of women who had given birth as an adolescent, and the likelihood of a girl having given birth as an adolescent. Both of the above mentioned interactions together (not shown) retained significance in a final model, with an AIC of 2606, a 13-point decrease from Model 2, including no interactions. This is evidence that both interactions in the model improve model fit.

#### 5.5.1 Sensitivity analyses

Some of the individual-level controls we used for this analysis, although potentially important confounders for the relationship between village-level factors and adolescent birth, could be the result of having given birth as an adolescent. Therefore, we ran our main model, Model 2, eliminating education, income, food security, time spent in the village, the individual injunctive norms, and the individual attitudes measures (See online appendix). Our results did not change. Because the attitudinal and normative questions regarding adolescent birth are phrased to be specific to births before age 18 years, we reran all of our models using giving birth before age 18 years as the measure for adolescent birth. Again, the results did not change, suggesting that the relationships we identified in these analyses are robust.

### 5.6 Spatial analyses

**Fig. 3:**
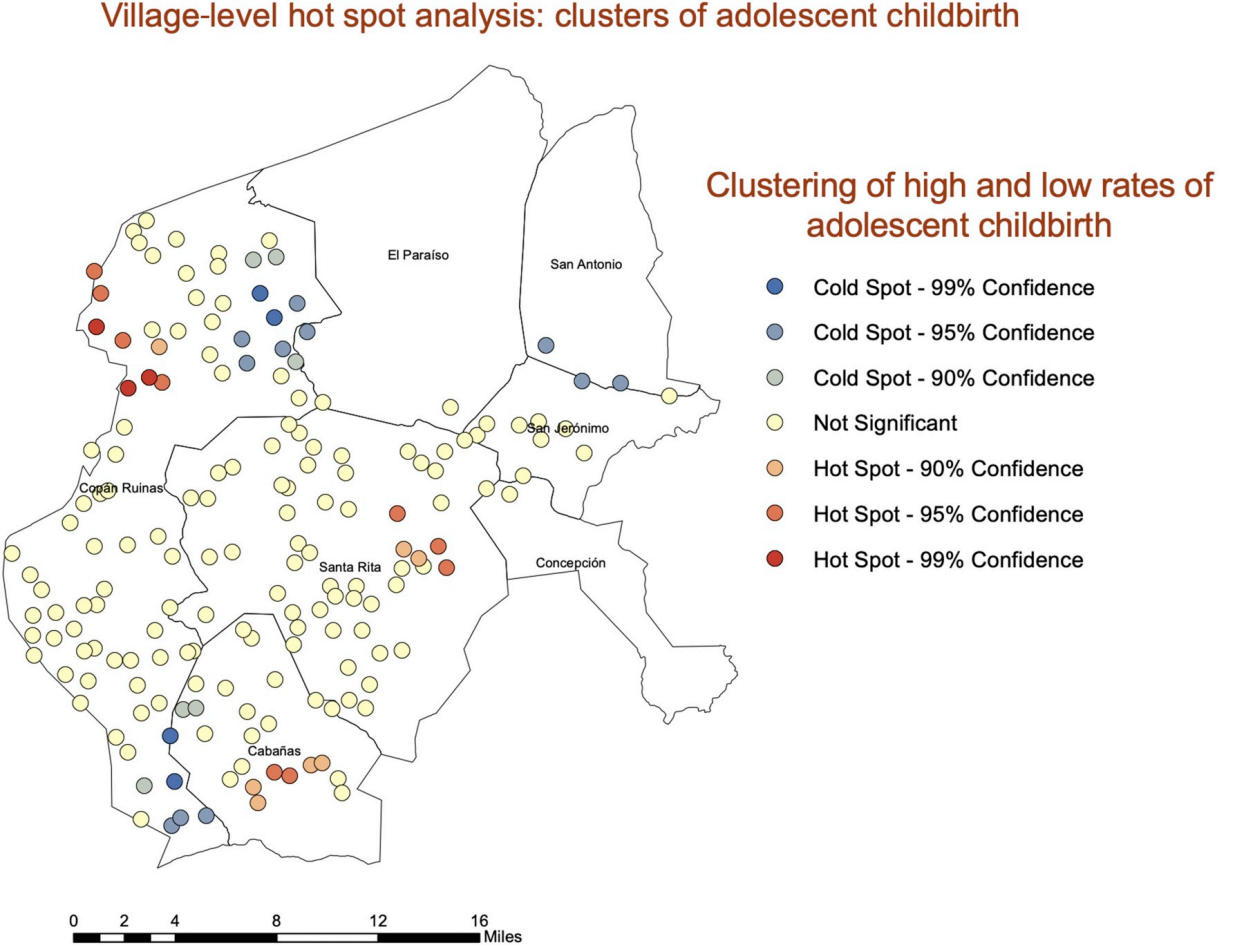
A hot spot analysis of adolescent births at the village level, limited to women younger than 21 years.

**Fig. 4:**
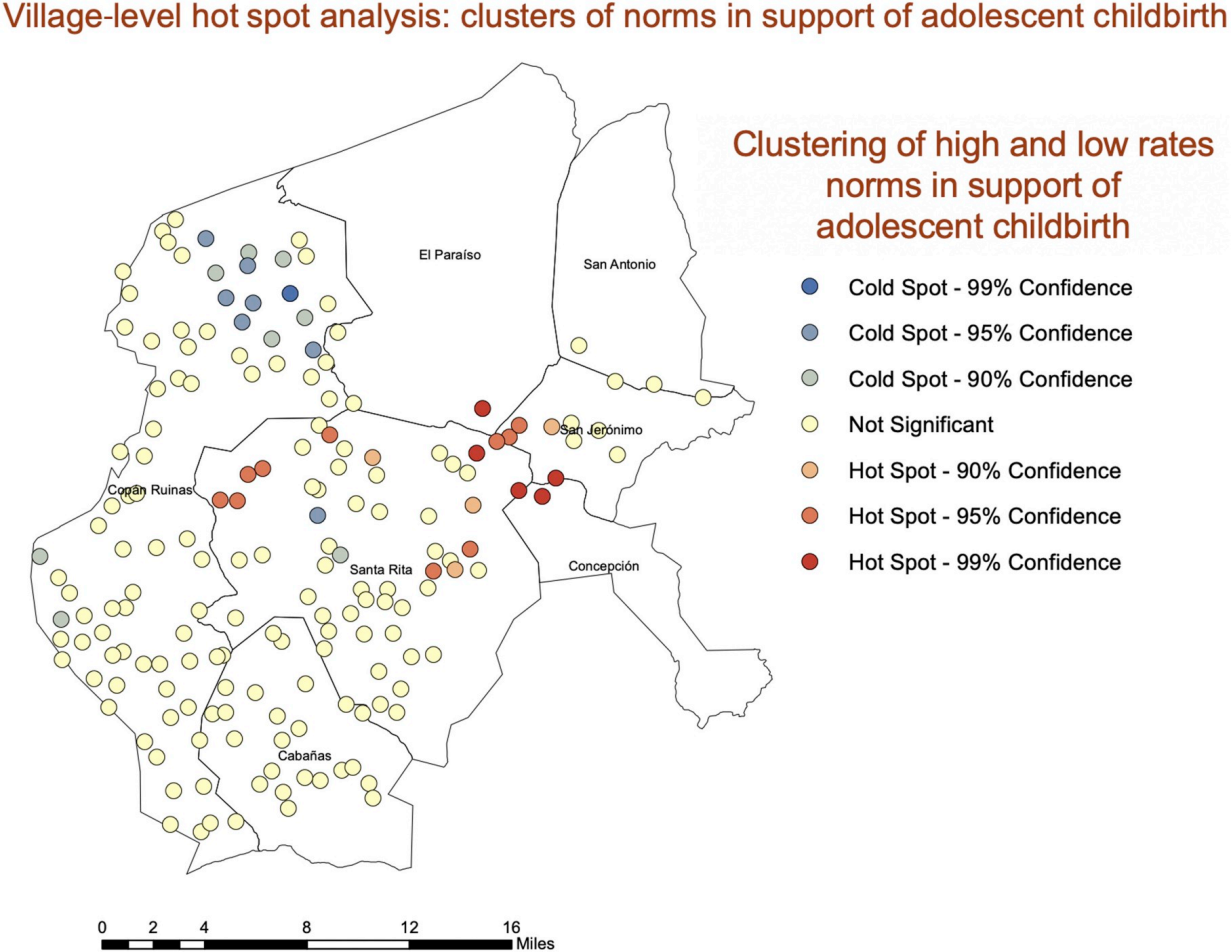
A hot spot analysis of norms in favor of adolescent birth at the village level.

A high value (hot spot) indicates that a village is surrounded by other villages with high values, whereas a low value (cold spot) indicates that a village is surrounded by other villages with low values. Fig. 3 shows the clustering of adolescent births among the study villages, and Fig. 4 shows the clustering of perceived normative approval of adolescent birth among the study villages. Both variables exhibit spatial clustering, indicating the existence of statistically significant spatial dependence in the data.

**Fig. 5:**
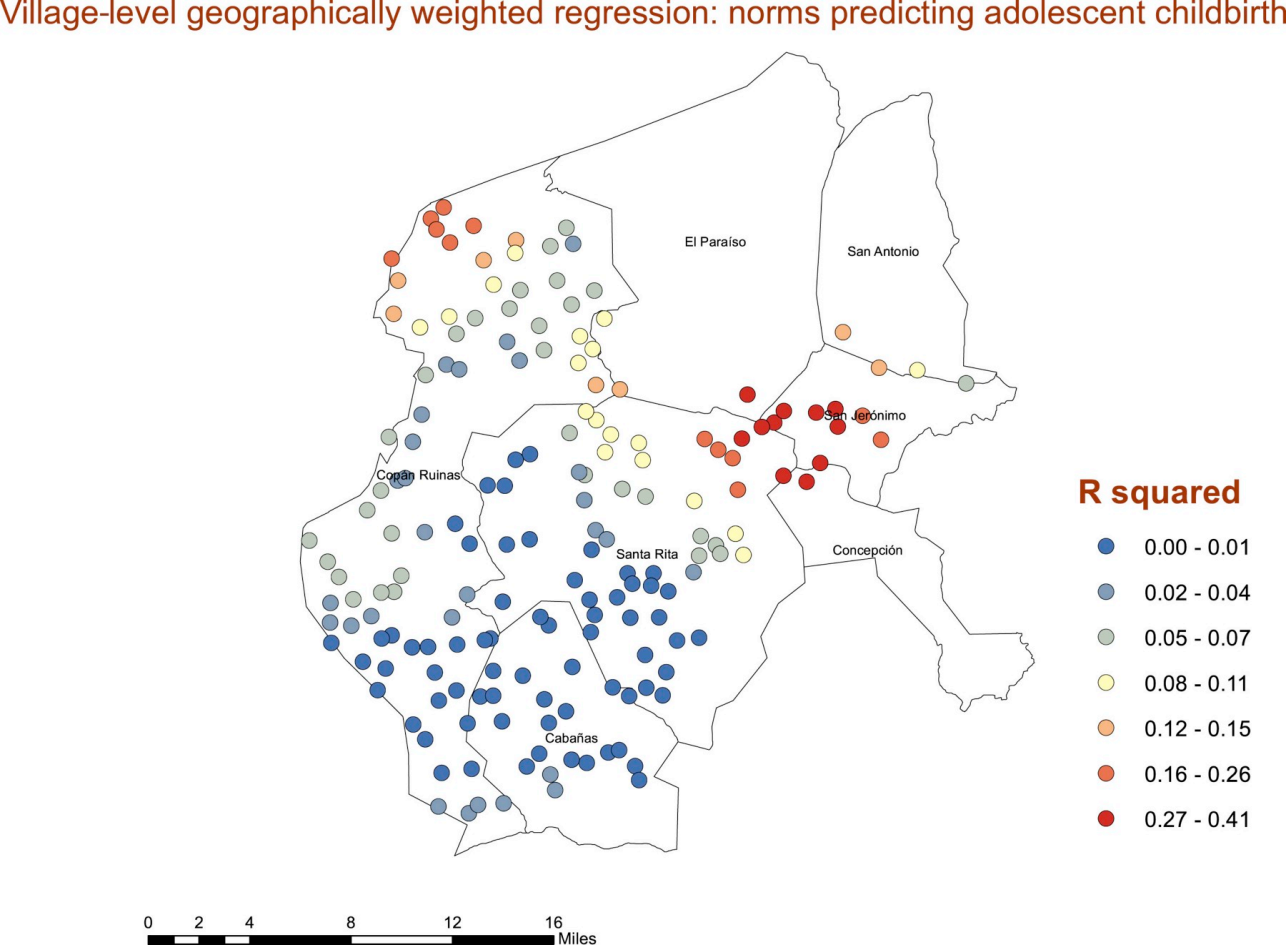
Geographic variation in the R squared values in a linear model with proportion of village in support of adolescent birth predicting village level proportion of women younger than 21 years who have had an adolescent pregnancy. Red nodes are those with higher R squared values, suggesting the association is stronger in those areas.

Using the proportion of women between the ages of 15 and 20 years who gave birth as adolescents as our outcome variable and the proportion of village respondents who report positive community norms regarding adolescent birth as the main predictor, we found significant geographic variation in the statistical association of normative beliefs and adolescent birth at the village level. Rather, the relationship was consistently strong throughout the department. Generally, where there are hot and cold spots of norms in support of adolescent childbirth, the association between village-level norms and village-level childbirth is the highest (see Fig. 5).

The next step in the spatial analysis was to adjust for the fact that hot spot analyses assess the spatial clustering of single variables but cannot incorporate multiple variables and their associations. Therefore, we used ArcGIS to run a grouping analysis—a type of cluster analysis that takes the spatial distribution of multiple variables into account. This allowed us to create “neighborhoods” of villages exhibiting similar levels of both adolescent birth and normative beliefs in support of adolescent birth. The Calinski-Harabasz pseudo F-statistic, which is a ratio reflecting within-group similarity and between-group difference, suggested that four groups would be optimal.

**Fig. 6:**
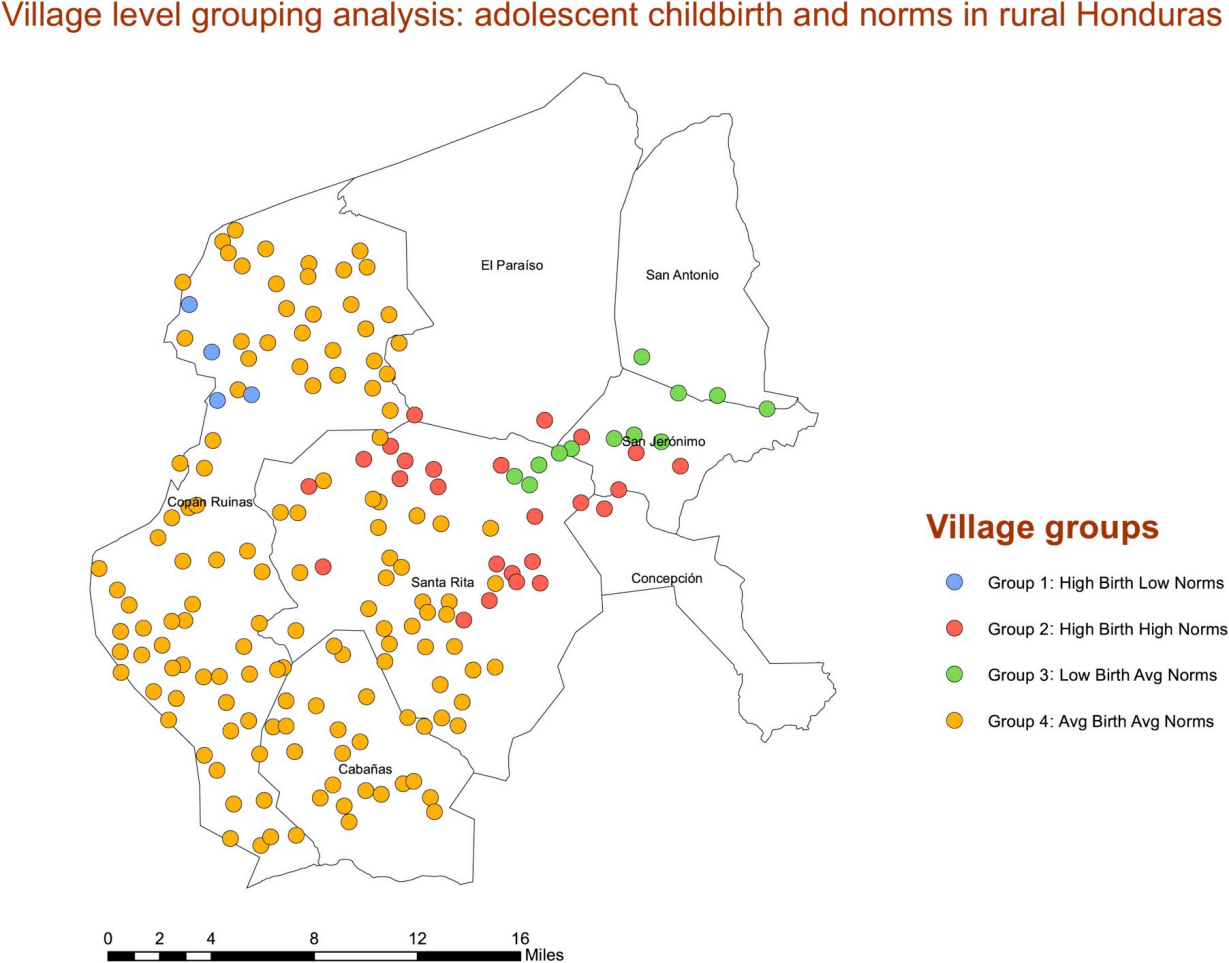
Geographic clustering of adolescent births and normative beliefs in support of adolescent births at the village level. The orange group is characterized by average levels of adolescent births and average levels of normative beliefs. Green is low levels of adolescent births along with average levels of norms in support of it. Red is characterized by higher levels of both. Finally, blue is an outlier, with average normative levels but higher rates of adolescent birth.

Fig. 6 shows the results of the grouping analysis that includes both village-level adolescent birth and village-level normative beliefs. The largest group in the map, colored in orange (N = 135), is characterized by average levels of adolescent birth and average levels of normative beliefs. The red group (N = 25) is characterized by higher levels of both. The green group (N = 12) consists of average norms and low adolescent birth. Finally, the blue group is an outlier (N = 4), with average normative levels but higher rates of adolescent births.

## 6 Discussion

In this analysis, we explored an unusual data set from rural Honduras that included demographic, behavioral, attitudinal, normative, and social network measures for a full population from 176 villages to consider the village, network, and normative predictors of adolescent birth among women between the ages of 15 and 20 years at the time of the survey. We predicted that village-level social network characteristics and village-level normative characteristics would be independently associated with the likelihood that a girl gave birth as an adolescent, and that these associations would be geographically clustered. Our hypotheses were partly borne out, in ways that can provide important insights for intervention efforts to reduce adolescent fertility in similar communities in Central America.

We expected to find that social cohesion, as measured by social network density, would be significantly associated with a decreased risk of adolescent birth, by virtue of implied higher levels of social surveillance. Because adolescent birth is associated with social instability and lower levels of social control (Denner et al., 2001), more-cohesive communities would potentially exert a protective effect, but we did not find this. We found that both descriptive and injunctive norms at the village level (collective norms and collective adolescent births) were strongly associated with adolescent birth and that this association was approximately one-third of what we found for education, one of the strongest and most consistent predictors of adolescent childbearing. We also found that there were differential effects on these normative associations, depending on village-level density and proportion of life spent in the village. It was not whether the aggregate of individuals in the community had individual beliefs about the appropriate age for a first birth, as the measure of collective attitudes was not statistically significant; rather, it was how those individuals collectively believed that others in the community would respond to such an event. A woman's own attitude regarding the appropriate age for a first birth was strongly associated with having given birth as an adolescent, and although a woman's own reported beliefs regarding community support for giving birth before age 18 years were associated with her likelihood of having done so, this effect was partially attenuated when we included village-level aggregate norms in the model. Of course, we can make no causal assumptions from a girl's own attitudes and normative perceptions. If she gave birth as an adolescent, she may have changed her beliefs in response. However, it is unlikely that the entire village would change its beliefs around adolescent births in response to one girl's birth, and one girls birth would not change the proportion of women who gave birth as adolescents. Although the time spent in the village could be a strong factor associated with village level factors and the probability of adolescent birth, most girls had been in the villages since birth, and our models accounted for this potentially confounding factor.

The fact that network density changes the association between collective norms and adolescent births is strong evidence that there may be causal connections in these dynamics. Injunctive norms work through direct social influence. The stronger the network, the stronger the influence. Highly dense networks reinforce and transmit norms much more than loosely connected networks (Haynie, 2001; Lin, 2017, pp. 3–28; Shakya, Christakis, & Fowler, 2014). Although this could be an artifact of village size, as smaller villages are naturally more dense, we controlled for this in the models. The fact that the association between descriptive norms (collective adolescent births) and adolescent births did not vary by level of village density is further evidence of a strong normative dynamic around adolescent birth. Descriptive norms are expected to influence behavior through social learning (Kohler, Berman, & Watkins, 2001). Individuals observe behavior, and if they conclude it is beneficial in some way, they may emulate it. This can come without social pressure, however, so the level of connection between individuals may be irrelevant. We found that the association between descriptive norms and adolescent births varied by time spent in the village, an indication that the longer the period of observation, the stronger the likelihood of emulating what is observed. These results have important implications for efforts to decrease adolescent childbearing in Central America, suggesting that the most important social context that increases adolescent births is normative in the injunctive and the descriptive sense, especially in tightly connected village contexts.

Consistent with the consideration that village-level normative factors may be an important factor underlying adolescent births in these contexts, our hot spot analysis and spatial grouping analyses showed significant geographic clustering of adolescent births and village-level norms—both hot spots and cold spots. Particularly noteworthy is the strong geographic cluster of high normative and high fertility villages in the eastern edge of our study region. This is important evidence that although norms and their associations are occurring at the village level, these associations are also occurring in geographic space. The fact that our geographically weighted regression showed spatial differences in the associations is also telling. Normative influence seems to be occurring where there are hot spots in norms and hot spots in adolescent childbearing rates. Understanding these contextual differences can be a crucial element to successful interventions to prevent adolescent childbearing in similar settings. Geographic clustering of norms and adolescent childbearing suggest that a blanket approach across departments could be ineffective. Understanding where there are clusters of risk both geographically and socially, and how to identify those clusters, can help interventionists target them far more effectively.

There are limitations to these analyses. First, the question about age at first birth is only asked of women who reported a live birth, so our estimates are likely to underestimate the earliest ages at which girls are becoming pregnant. Because these are cross-sectional data, we cannot track time-dependent associations between our predictors of interest and adolescent births. All questions are based on self-report so there is the possibility of response bias in some of our measures. It is also important to recognize that women were asked about their attitudes (and their view of community attitudes) after their first birth, so it is impossible to disentangle cause and effect. For some women, giving birth as an adolescent may be consistent with their prechildbearing views, whereas other women may be justifying their behavior after the fact. It is possible that our findings could be skewed by omitted variable bias. Village-level clustering of adolescent childbearing and its association with social norms may be driven by village-level factors for which we have no measures. Our future research will address these issues, as well as take a deeper look at the individual social dynamics through individual network analysis.

Despite these limitations, these data provide a rare opportunity to analyze a full census of a population, including a detailed reproductive history. The magnitude and consistency of our results suggest that adolescent fertility among girls in these populations is not only a common and serious issue but also depends on collective pressures organized in geographic and social space. Individual-level risk factors of adolescent fertility are fairly well established, and, in our analyses, we find individual-level results consistent with previous research. However, after controlling for these individual attributes, we still find variation in adolescent fertility rates. What does that variation come from? Our results show it is a combination of social normative and social network effects. Although social normative effects are the most strongly associated and are clustered geographically, village-level network patterns play an important role in moderating normative effects. In sum, individual attributes, geography, social norms, and social interactions all play roles in explaining variation in adolescent fertility in rural Honduras.

## Ethics

The Yale IRB and the Honduran Ministry of Health approved all data collection procedures (Protocol number 1506016012) and all participants provided informed consent before enrolment.

## Funding

Funding provided by the Bill and Melinda Gates Foundation, grant number OPP1098684, and NICHD grant number K01HD087551.

## Declaration of competing interest

The authors have no conflicts of interest or financial disclosures to report.
